# Simultaneous bicompartmental bucket handle meniscal tears with a clinically competent Anterior Cruciate Ligament

**DOI:** 10.1186/1749-799X-5-68

**Published:** 2010-09-15

**Authors:** Jonathan Wright, Chiharu Tamura, Iain Findlay, Aria Daneshfar

**Affiliations:** 1 CT1 Orthopaedics, University Hospital Lewisham, London, UK; 2 Orthopaedic SpR, University Hospital Lewisham, London, UK; 3 Consultant Orthopaedic Surgeon, University Hospital Lewisham, London, UK

## Abstract

Bucket handle meniscal tears (BHMT) of the knee occur infrequently (approximately 10% of meniscal injuries). Simultaneous, bicompartmental BHMT are extremely rare. Previously, these have only been reported in association with a ruptured anterior cruciate ligament (ACL). The pathomechanism of this injury was thought to be due to the lack of knee stability following the ACL injury. We present a case of a 38 year old male patient with bicompartmental BHMT with a clinically competent ACL. This highlights the need for clinical and radiological suspicion of simultaneous BHMTs even in the presence of an intact ACL.

## Background

Knee menisci are important structures in load transmission, shock absorption and joint stabilization[1]. Injuries to the menisci often result from a forceful twisting movement of the knee whilst weight bearing. A bucket handle meniscal tear (BHMT) is a sub group of the meniscal injuries, which consist of a vertical or oblique longitudinal tear with an attached fragment displaced away from the periphery of the meniscus [2]. BHMT occurs more commonly in the less mobile, medial meniscus [2]. There have been 3 case reports of simultaneous bicompartmental BHMT all of which were associated with torn ACL [3-5]. We present an unusual pattern of this meniscal injury: bicompartmental BHMT with a clinically competent ACL.

## Case History

A 38 year old man presented with persistent instability and swelling to his left knee following a twisting injury, whilst dancing. There was no previous history of injury or knee symptoms.

On examination, he was tender along the medial and lateral joint lines. His ACL was clinically intact and Mc Murray's test was positive for both menisci.

**Figure 1:**
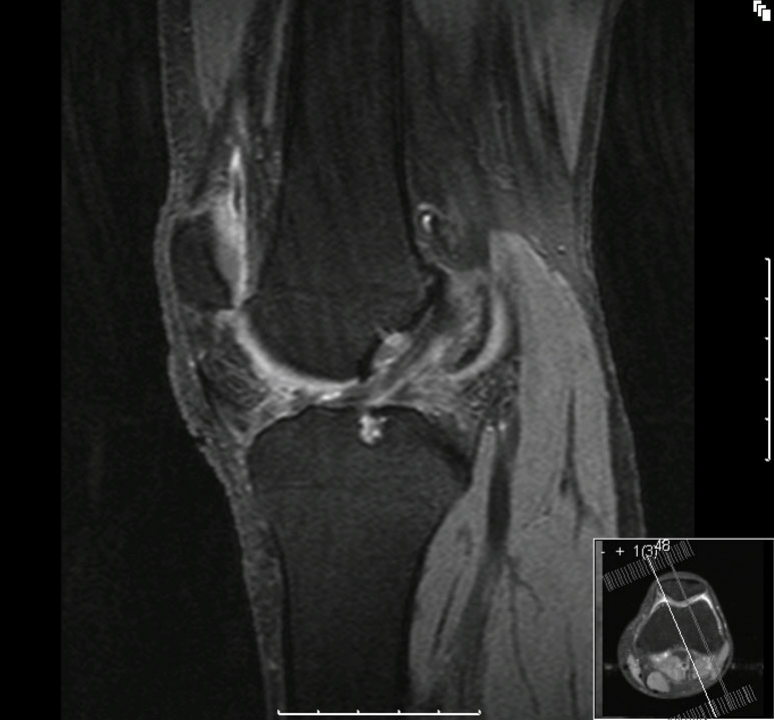
**T2 weighted MRI image demonstrating intact anterior cruciate ligament**.

**Figure 2:**
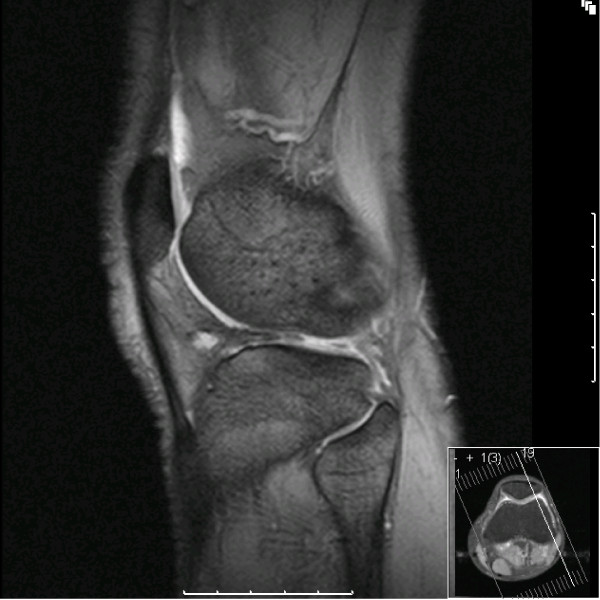
**T2 weighted MRI image demonstrating lateral meniscal tear; "Absent bow tie sign"**.

MRI of the left knee showed features of a bucket handle tear in both medial and lateral meniscus with an intact ACL (Figure 1, 2).

**Figure 3:**
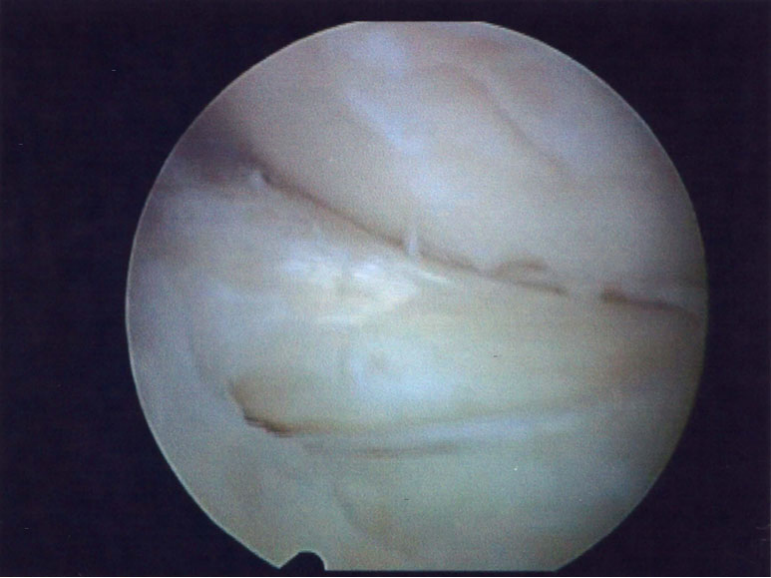
**Arthroscopic images of medial meniscus bucket handle tear**.

**Figure 4:**
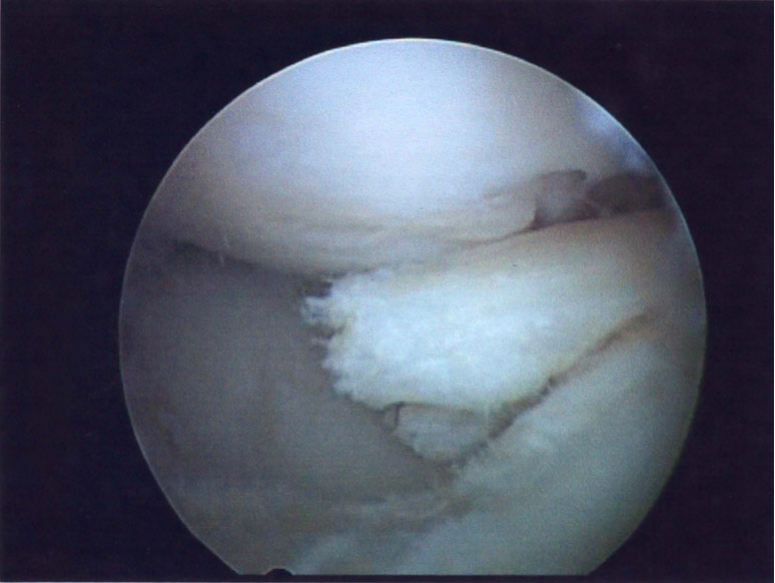
**Image of arthroscopic debridement of lateral meniscal tear**.

He underwent left knee arthroscopy, which confirmed bucket handle meniscal tears in both medial and lateral compartment and 50%, partial rupture of ACL. Examination under anaesthesia demonstrated clinical competence of the ACL. Partial meniscectomy in both compartments was performed (Figures 3, 4).

He was reviewed in the clinic 6 weeks after the arthroscopy and reported an uneventful recovery.

## Discussion

In BHMTs, a longitudinal split extending from the posterior horn of the meniscus to the anterior horn allows the inner segment to displace and this resembles a handle. The non displaced portion of the meniscus has the appearance of a bucket[6].

BHMTs are reported to occur in approximately 9-24% of meniscal lesions[7]. Only three cases of bicompartmental BHMT have been reported in the past, all of which were associated with ACL tears [3-5]. These lesions typically occur in young age group, usually following a significant trauma with sudden impact to split the meniscus [3]. There is a significant male preponderance for the occurrence of meniscal bucket-handle tears [8] and three times more in the medial menisci compared to the lateral menisci [2] as the medial meniscus is less mobile than the lateral meniscus[5].

Clinically, patients may present with a lack of full extension, history of knee locking or completely locked knee [9,10]. The locked knee occurs in medial BHMTs as well as in the lateral BHMT with similar percentage [10].

Two main modalities of investigation are MRI and knee arthroscopy. Overall, sensitivity and positive predictive value of MR imaging for the detection of meniscal bucket-handle tears were calculated as 90% [8]. There are several signs of BHMT described on MRI including absent bow tie signs, flipped meniscal signs or double delta sign and double PCL in sagittal views, coronal truncation sign and fragment in intercondylar notch in coronal views [1-3,6].

The menisci, in particular the medial, provide a role in stability of the knee particularly in association with ACL deficiency. This is an important consideration as the previous reports of bicompartmental BHMT have all been associated with ACL deficiency. The forces through the medial meniscus have been shown to increase by 197% at 60 degrees of flexion following loss of the ACL [11]. Cadaveric studies have demonstrated significantly increased antero-posterior tibial translation following partial or total medial meniscectomy in the ACL deficient knee, while the stability is not affected if the ACL remains intact [12,13]. The lateral meniscus has less contribution to stability, with little increase in tibial translation following meniscectomy [14].

Our patient underwent arthroscopic meniscectomy. One of the previously reported cases of bicompartmental BHMT with ACL deficiency offered a partial meniscectomy and arthroscopically assisted ACL reconstruction with bone-patellar reconstruction, as the tears were not reparable[5]. The meniscal lesions could be managed by reparative surgery if there is a potential to heal post operatively. Thus, factors to consider for repair operation are: acute injury, rather than degenerative, size of the lesion and vascular supply to the affected part of menisci (the closer the lesion to the meniscosynovial junction, the better the vascularization) [1].

Our case highlights the need for clinical and radiological suspicion of simultaneous bicompartmental bucket handle tears even in the presence of an intact ACL and without a history of significant trauma.

## Consent

Written informed consent was obtained from the patient for publication of this case report and accompanying images. A copy of the written consent is available for review by the Editor-in-Chief of this journal.

## Competing interests

The authors declare that they have no competing interests

## Authors' contributions

JW and CT performed the literature search and drafted the article. JW performed the subsequent revisions. IF conceived the article and provided guidance on design and corrections. AD supervised, co-ordinated and provided further advice on revisions. All authors read and approved the final manuscript.
